# Changes in gut microbiota and plasma inflammatory factors across the stages of colorectal tumorigenesis: a case-control study

**DOI:** 10.1186/s12866-018-1232-6

**Published:** 2018-08-29

**Authors:** Yongzhen Zhang, Xin Yu, Enda Yu, Na Wang, Quancai Cai, Qun Shuai, Feihu Yan, Lufang Jiang, Hexing Wang, Jianxiang Liu, Yue Chen, Zhaoshen Li, Qingwu Jiang

**Affiliations:** 1 Department of Gastroenterology, Changhai Hospital, Naval Medical University, 168 Changhai Road, Shanghai, 200433 China; 2 The 92914th Military Hospital of PLA, Shanghai, China; 3 Key Laboratory of Public Health Safety of Ministry of Education, School of Public Health, Fudan University, 130 Dong’an Road, Shanghai, 200032 China; 4 Department of General Surgery, Changhai Hospital, Naval Medical University, Shanghai, 200032 China; 5 School of Epidemiology and Public Health, University of Ottawa Faculty of Medicine, Ottawa, Canada

**Keywords:** Colorectal cancer (CRC), Gut microbiota, Plasma inflammatory factors, Correlation analysis

## Abstract

**Background:**

Colorectal cancer (CRC) is a common malignant gastrointestinal tumor. In China, CRC is the 5th most commonly diagnosed cancer. The vast majority of CRC cases are sporadic and evolve with the adenoma-carcinoma sequence. There is mounting evidence indicating that gut microbiota and inflammation play important roles in the development of CRC although study results are not entirely consistent. In the current study, we investigated the changes in the CRC-associated bacteria and plasma inflammatory factors and their relationships based on data from a case-control study of Han Chinese. We included 130 initially diagnosed CRC patients, 88 advanced colorectal adenoma patients (A-CRA), 62 patients with benign intestinal polyps and 130 controls.

**Results:**

Fecal microbiota composition was obtained using 16S ribosomal DNA (16S rDNA) sequencing. PCOA analysis showed structural differences in microbiota among the four study groups (*P =* 0.001, Unweighted Unifrac). Twenty-four CRC-associated bacteria were selected by a two-step statistical method and significant correlations were observed within these microbes. CRC-associated bacteria were found to change with the degree of malignancy. Plasma C-reactive protein (CRP) and soluble tumor necrosis factor II (sTNFR-II) displayed significant differences among the four study groups and increased with adenoma-carcinoma sequence. The correlations of CRP and sTNFR-II with several CRC-associated microbes were also explored.

**Conclusions:**

CRC-associated species and plasma inflammatory factors tended to change along the adenoma-carcinoma sequence. Several CRC-associated bacteria were correlated with CRP and sTNFR-II. It is likely that gut microbiome and inflammation gradually form a microenvironment that is associated with CRC development.

**Electronic supplementary material:**

The online version of this article (10.1186/s12866-018-1232-6) contains supplementary material, which is available to authorized users.

## Background

Colorectal cancer (CRC) is the third common cancer worldwide causing 1.4 million newly diagnosed cases and 694,000 deaths a year [1]. In China, CRC is the 5th most commonly diagnosed cancer for both males and females and the incidence rate has been rapidly increasing recently [2].

The development of CRC is regarded as a multifactorial process involving genes mutation accumulation, inflammation and lifestyle factors, such as dietary habits and smoking [3–6]. Gut microbiome and inflammation are hypothesized to shape the tumor microenvironment and promote the tumorigenesis [7–10]. Previous observational and experimental studies have identified and suspected several microbes as potential drivers of CRC, including *Fusobacterium nucleatum*, *Streptococcus bovis/gallolyticus*, enterotoxigenic *B. fragilis* (ETBF), *Enterococcus faecalis* and colibactin-producing *Escherichia coli.* In recent studies, more detailed microbial profiles gained by high-throughput analyses, like metagenomic shotgun-sequencing and 16S rDNA sequencing, have revealed more bacteria that are associated with CRC [11–13].

Pathogenic microbes may work with inflammatory factors in CRC progression [14]. The enrichment of *S. bovis* is associated with an increased expression of pro-inflammatory genes [15]. ETBF can activate the signal transducer and activator of transcription 3 (STAT3) and induce Th17 cell infiltration as well as cytokines releasing in the colon of ApcMin/+ mice [5]. *F. nucleatum* infection can increase the expression of proinflammatory genes such as Scyb1, Interleukin-6 (IL-6), tumor necrosis factor (TNF-α), and Mmp3 [16]. However, there is a lack of consistent results for the effects of plasma inflammatory factors on CRC development from population-based studies [14, 17–21].

The major course of sporadic CRC progression begins with aberrant crypts, advances with early and late adenomatous polyps and finally turns into invasive carcinoma [6]. However, the changes in CRC-associated bacteria and inflammatory factors across the adenoma-carcinoma sequence and the potential correlations between them have not been clarified. We conducted a case-control study, which included CRC patients, A-CRA patients, patients with benign polyps and controls, to examine the relationships among CRC-associated microbiota, inflammatory factors and colon cancer status.

## Methods

### Study population and sampling

A case-control study was carried out and participants were from outpatients who received the colonoscopy at Changhai Hospital in Shanghai in 2014-2015. Persons who were capable to complete a questionnaire interview and to provide relevant biological samples were eligible for study inclusion.

Patient exclusion criteria included: (a) patients younger than 40 years of age, (b) persons not Han people, (c) patients with prior diagnoses of colorectal cancer, colorectal adenoma, inflammatory bowel disease (IBD) or other cancers, (d) patients had a family history of colorectal cancer in first- and second-degree relatives and no family history of neoplastic polyps or hereditary syndromes in first degree relatives under 60 years of age, and (e) patients had used antibiotics in last 6 months before colonoscopy. Hereditary syndromes include familial adenomatous polyposis (FAP), hereditary nonpolyposis colorectal cancer (HNPCC), Turcot syndrome, Oldfield syndrome and juvenile polyposis syndrome. Control selection was based on the same inclusion/exclusion criteria as used for case selection.

All participants had not been diagnosed or screened positive for colorectal cancer before inclusion and had no diet restrictions. Controls were outpatients who had no CRC or polyps indicated by colonoscopy and had no specific symptoms of CRC and were frequently matched with CRC patients by gender and age. Polyethylene glycol lavage solution was used for bowel preparation. Colonoscopy was performed by experienced endoscopists using a standard video colonoscope (Olympus Optical Co, Tokyo, Japan). Written informed consents were acquired from all participants.

### Sample collection and laboratory testing

Before the colonoscopy, fresh stool samples (≥1 g) were collected at the hospital in a unified large centrifuge tube and stored in the − 80 °C fridge instantly. The stool samples were frozen-thawed once before an extraction of DNA. Biopsy specimens were collected during colonoscopy. After the colonoscopy, a questionnaire interview was conducted to collect basic demographic information, medical history and lifestyle factors (including smoking and alcohol drinking). Participants provided 2 ml venous blood that was preserved in − 80 °C fridge instantly for corresponding laboratory assay. Blood samples were centrifuged at 3000 rpm for 10 min to separate plasma. Plasma soluble tumor necrosis factor receptor 2 (sTNFR-II) was measured as a surrogate for TNF-α. CRP, IL-6, and sTNFR-II in plasma were measured with ELISA method (human CRP ELISA Kit 96 T, Anogen; Human IL-6 ELISA Kit 96 T, Anogen; Human sTNFR-II ELISA Kit 96 T, Raybiotech) according to standard procedures provided by the manufacturers.

Obesity/overweight status was assessed according to the criteria for Chinese adults (defines overweight as BMI ≥ 24 kg/m^2^ and obesity as BMI ≥ 28 kg/m^2^) [22]. For the patients, lesions located in the cecum, ascending colon, hepatic flexure, transverse colon and splenic flexure were considered as proximal lesions, and descending colon, sigmoid colon and rectum were deemed distal lesions [23].

### DNA extraction and 16S rDNA sequencing

Bacterial genomic DNA was extracted from stool samples using OMEGA-soil DNA Kit (USA Omega Bio-Tek) and examined by 1% agarose gel electrophoresis. V3-V4 region of the 16S rDNA gene was amplified by universal primers (forward 338F 5’-ACTCCTACGGGAGGCAGCAG-3′ and reverse 806R 5’-GGACTACHVGGGTWTCTAAT-3′), while attaching Illumina adapters and sample-specific barcode sequences. The V3-V4 hypervariable region provides appropriate information for taxonomic classification of microbial analysis from specimens associated with human microbiome studies and was used by the Human Microbiome Project [24]. Polymerase chain reaction (PCR) was performed by ABI GeneAmp® 9700, with TransStart Fastpfu DNA Polymerase, 20 μl reaction systems. Each sample repeated the conduction for three times and PCR products, quantified by QuantiFluor™ -ST Fluorescence System (Promega), was pooled by AxyPrep DNA gel extraction kit (Axygen, USA) to prepare for sequencing. The Illumina MiSeq platform (Illumina, USA) was served in sequencing the amplicons.

### Bioinformation analysis and statistical analysis

Paired-end reads were merged into one sequence in terms of overlapping (FLASH) and split by barcodes and primers. Reads with low quality and adaptors were removed (Trimmomatic 0.27). Dereplicated sequences (without singletons) were clustered into operational taxonomic units (OTUs), with 97% similarity. Representative sequences were picked out and classified by SINTAX (USEARCH v.10) algorithm using the RDP training set v16 with species names at a confidence threshold of 0.8 for genus and of 0.5 for species [25].

Alpha-diversity and the β-diversity of the microbiota data were computed using QIIME v.1.9.1 to assess the diversity alteration of microbiome [26]. Alpha-diversity measurements included ace, shannon index, simpson and PD whole tree. β-diversity was measured by unweighted and weighted UniFrac distances between samples considered phylogenetic information. To assess overall fecal microbiota composition discrepancies, Permutational Multivariate Analysis of Variance Using Distance Matrices (PMANOVA) was performed based on the UniFrac distances, and potential confounders (gender, age, BMI, smoking and drinking status) were taken into consideration. Principal Coordinates Analysis (PCoA) was applied to visualize similarities or dissimilarities of the microbiota of samples in different groups using the β-diversity distances mentioned previously.

CRC-associated microbes were selected by two steps. Firstly, species were applied the Zero-inflated Log-Normal mixture model in the metagenomeSeq packages and microbes with adjusted *P* values less than 0.05 were selected [27]. Secondly, selected microbiota was further filtered by the random forest algorithm in the Boruta package with 1000 iterations [28]. Selected species were clustered by a hierarchical ward-linkage method with Spearman correlation. *P* values of multiple comparisons or correlations tests were adjusted using the Benjamini-Hochberg method.

Pearson χ^2^ test or Fisher’s exact test was applied to analyze qualitative clinical information of patients if appropriate. One-way ANOVA followed by Tukey HSD test was used to analyze the differences in age and α-diversity among the four study groups. Kruskal-Wallis test and Dunn’s test post-hoc method was applied to assess the differences in inflammatory factors. Jonckheere-Terpstra test was used to investigate the trend for inflammatory factors and CRC-associated bacteria along with the adenoma-carcinoma sequence. Correlation networks based on Spearman’s rank correlations were performed to visualize the associations between serum factors and gut microbiota by Cytoscape 3.6.1. Statistical analyses were carried out in R v 3.4.4.

## Results

### Demographic and clinical information

**Fig. 1:**
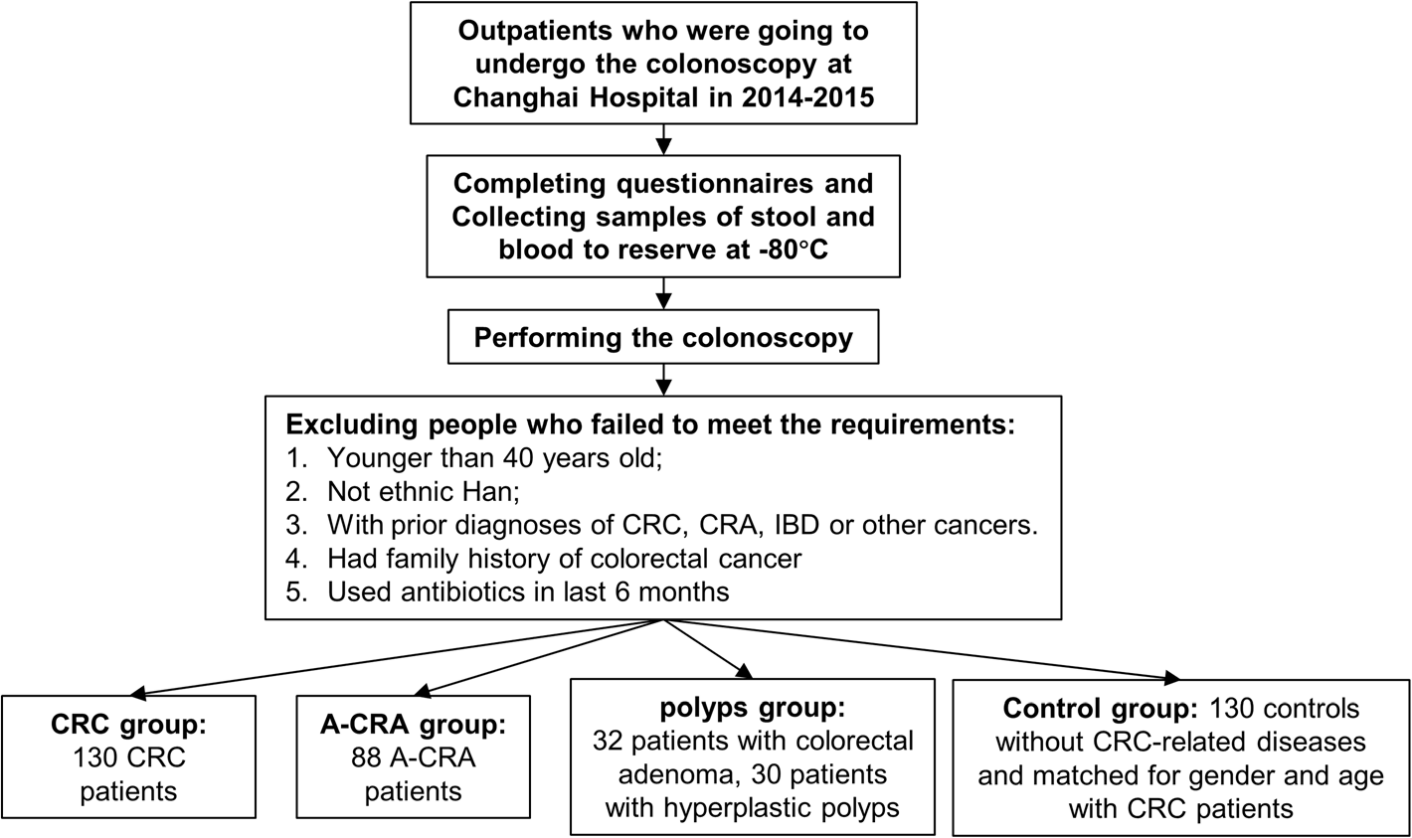
Flowchart for the selection of participants and collection of specimens. Gender, BMI and chronic diseases (heart disease, hypertension, and diabetes) showed no significant differences among the four study groups. Mean (SD) age of all participants was 59.1 (9.6) years. The included patients in the polyps group were younger than those with CRC (*P* = 0.033). The proportion of smoking in CRC (*P* = 0.044) and A-CRA (*P* = 0.002) patients was higher than controls. There were more patients with distal lesions than those with proximal lesions (Table 1). Among the CRC patients, 20.7%, 42.3% and 36.2% were in the TNM stages I, II and III, respectively

A total of 130 CRC patients, 88 A-CRA patients, 32 patients with colorectal adenoma, 30 patients with hyperplastic polyps and 130 controls were included in our study. We combined the colorectal adenoma patients and patients with hyperplastic polyps into one group (“polyps group”). The selection of participants and collection of specimens are summarized in Fig. 1.

**Table 1:** Demographic and clinical characteristics of patients and controls

	CRC (*n* = 130)	A-CRA (*n* = 88)	polyps (*n* = 62)	controls (*n* = 130)	*P* Value
Gender					
Male	65 (50.0%)	55 (62.5%)	31 (50.0%)	65 (50.0%)	0.228
Female	65 (50.0%)	33 (37.5%)	31 (50.0%)	65 (50.0%)	
Age(years)^a^	60.5 (9.8)	59.6 (10.3)	56.5 (8.9)	58.6 (8.9)	0.045
BMI (kg/m^2^)					
< 24.0	69 (53.1%)	44 (50.0%)	30 (48.4%)	74 (56.9%)	0.299
24.0-27.9	51 (39.2%)	34 (38.6%)	20 (32.3%)	43 (33.1%)	
≥ 28.0	10 (7.7%)	10 (11.4%)	12 (19.4%)	13 (10.0%)	
Alcohol drinking					
Never	98 (75.4%)	61 (69.3%)	50 (80.6%)	109 (83.8%)	0.069
Ever	32 (24.6%)	27 (30.7%)	12 (19.4%)	21 (16.2%)	
Smoking					
Never	91 (70.0%)	54 (61.4%)	42 (67.7%)	105 (80.8%)	0.015
Ever	39 (30.0%)	34 (38.6%)	20 (32.3%)	25 (19.2%)	
Lesion location					
Proximal	45 (34.6%)	27 (30.7%)	30 (48.4%)	–	0.072
Distal	85 (65.4%)	61 (69.3%)	32 (51.6%)	–	
Hypertension					
No	89 (68.5%)	58 (65.9%)	45 (72.6%)	88 (67.7%)	0.854
Yes	41 (31.5%)	30 (34.1%)	17 (27.4%)	42 (32.3%)	
Heart Disease					
No	122 (93.8%)	83 (94.3%)	59 (95.2%)	128 (98.5%)	0.223
Yes	8 (6.2%)	5 (5.7%)	3 (4.8%)	2 (1.5%)	
Diabetes					
No	117 (90.0%)	80 (90.9%)	59 (95.2%)	120 (92.3%)	0.658
Yes	13 (10.0%)	8 (9.1%)	3 (4.8%)	10 (7.7%)	

^a^Age was shown as mean (SD). *BMI* Body mass index

### Stool microbiota sequencing results

A total of 28,800,738 high-quality reads were obtained from 410 samples (mean = 70,245.7). We subsampled 29,719 reads for each participant according to the sample with the least sequences. An OTU table with 1794 OTUs was constructed based on these sequences. Among the OTUs, 7 phyla, 22 classes, 34 orders, 70 families, 163 genera and 285 species were assigned (inclusive conditions: phylum > 0.1%, class to species > 0.001%).

PCoA based on unweighted Unifrac distances showed a significant difference in gut microbiota among the four study groups (*P =* 0.001 for unweighted Unifrac distances; *P =* 0.320 for weighted Unifrac distances, PMANOVA, controlling for gender, age, BMI and status of smoking and drinking, Additional file 1: Figure S1). Alpha-diversity showed no significant difference among the four groups (ace: *P* = 0.153, shannon: *P* = 0.983; simpson: *P* = 0.814; PD whole tree: *P* = 0.08).

### CRC-associated microbiota in CRC patients compared with controls

**Fig. 2:**
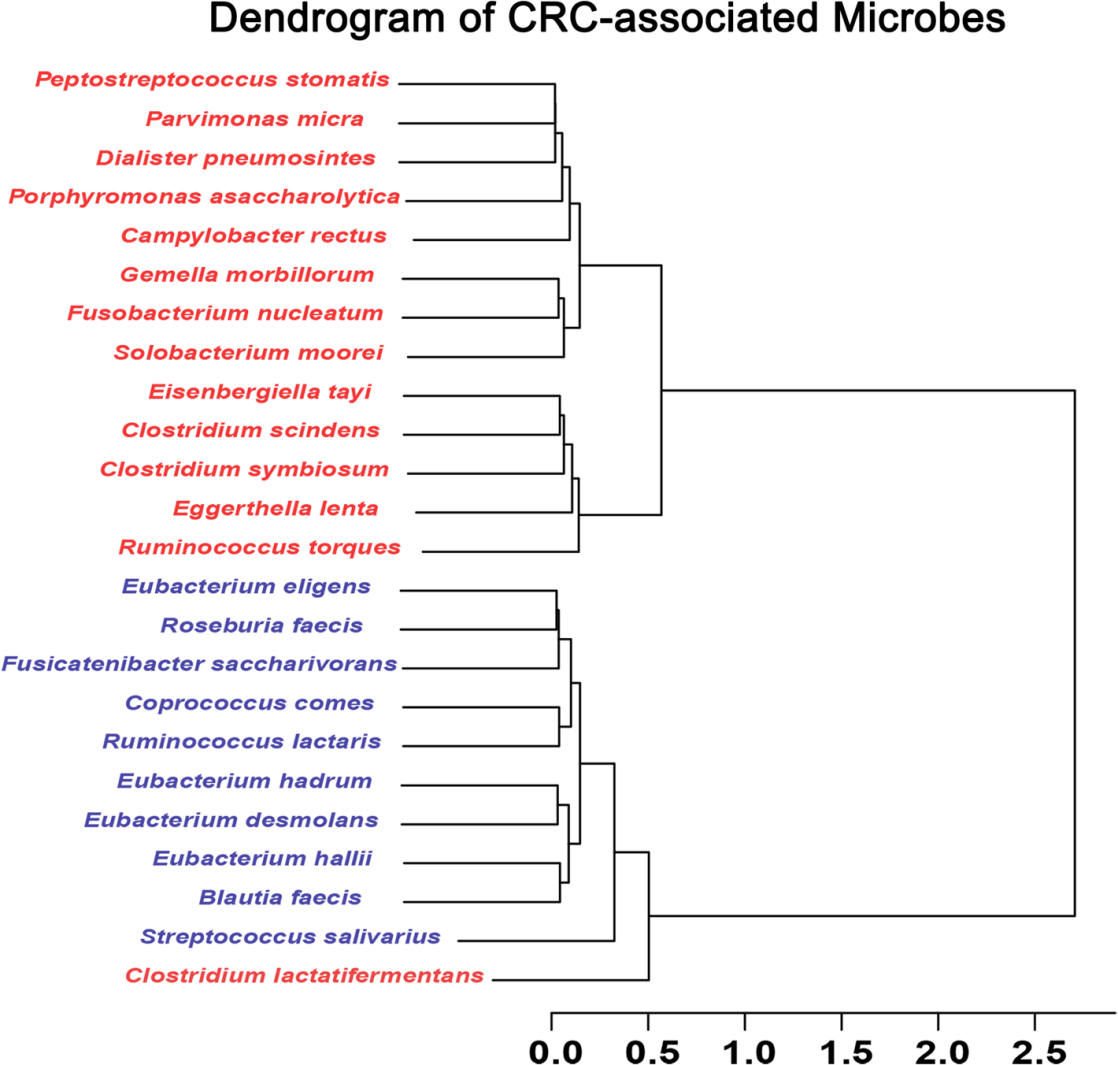
Hierarchical ward-linkage clustering of CRC-associated species. Red labels represent for microbes increased in CRC patients while blue for decreased. The clustering was based on Spearman’s correlations among the four study groups

**Fig. 3:**
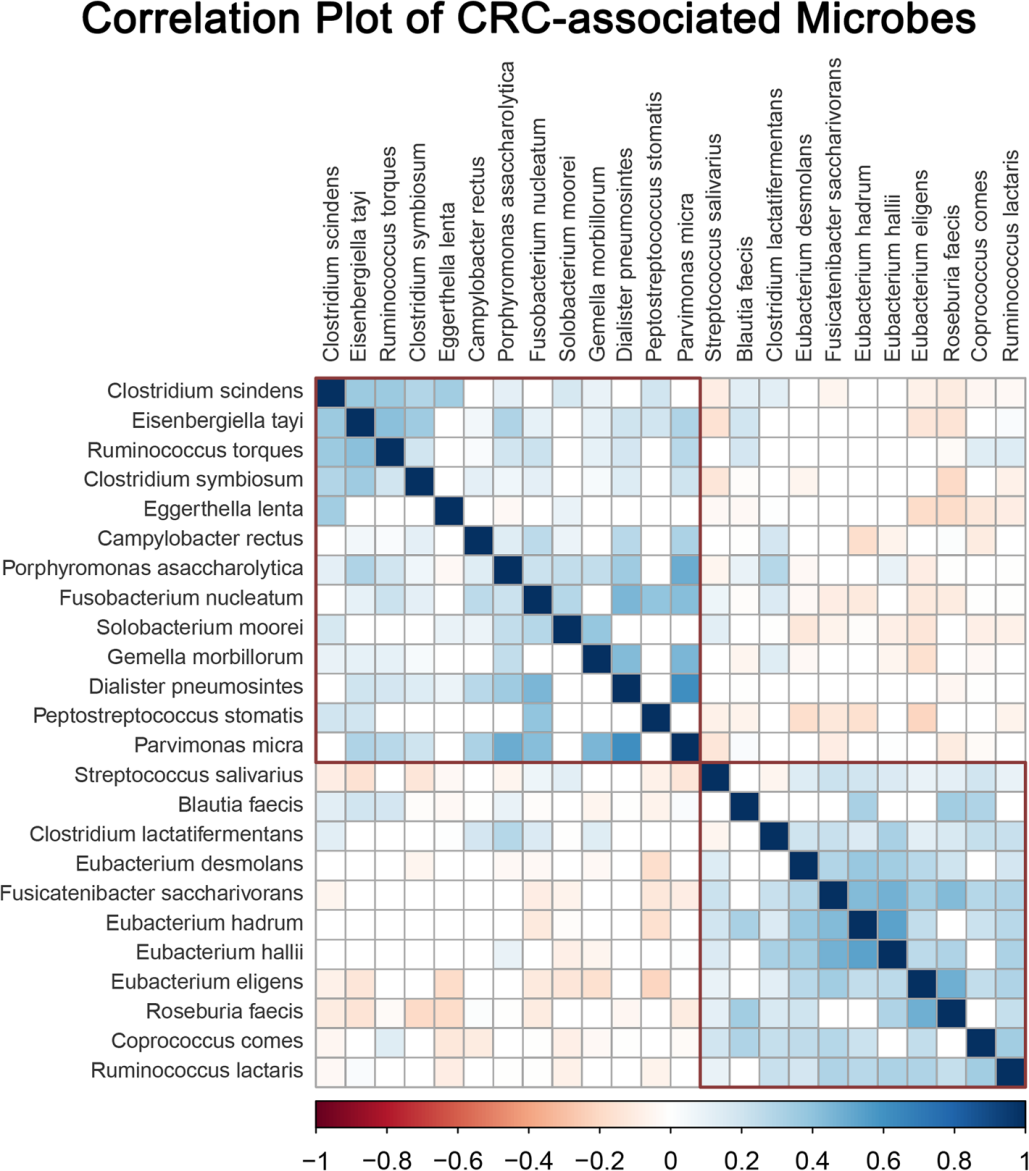
Correlation plot of CRC-associated microbiota in CRC patients compared with controls. Correlations with an adjusted *P* value less than 0.05 were displayed

CRC-associated microbiota at the species level was selected. A total of 24 species were identified and filtered by the Zero-inflated Log-Normal mixture model and random forest algorithm. The CRC-associated species were divided into two clusters according to the hierarchical ward-linkage clustering in all 410 participants (Fig. 2). Pairwise correlations displayed in Fig. 3 showed that species within the cluster were positively related. Fourteen of 24 species increased in CRC patients including *Peptostreptococcus stomatis*, *Parvimonas micra*, *Gemella morbillorum*, *Dialister pneumosintes*, *Porphyromonas asaccharolytica*, *Solobacterium moorei*, *Eisenbergiella tayi*, *Fusobacterium nucleatum*, *Ruminococcus torques*, *Eggerthella lenta*, *Clostridium symbiosum*, *Campylobacter rectus*, *Clostridium scindens*, *Clostridium lactatifermentans*. The other 10 species including *Eubacterium eligens*, *Coprococcus comes*, *Eubacterium hadrum*, *Eubacterium hallII*, *Fusicatenibacter saccharivorans*, *Blautia faecis*, *Roseburia faecis*, *Ruminococcus lactaris*, *Eubacterium desmolans*, *Streptococcus salivarius,* decreased in CRC patients (Additional file 2: Table S1).

### Results of inflammatory factors in plasma

CRP and sTNFR-II levels were significantly different in the four study groups. We discovered that the level of plasma CRP in CRC patients was higher compared with the A-CRA group (*P <* 0.001) and the control group (*P =* 0.002). The CRP level in the polyps group was higher than controls (*P* = 0.031). The plasma sTNFR-II in the CRC group (*P <* 0.001) and the A-CRA group (*P =* 0.001) was higher compared with controls.

### Trend analyses of CRC-associated bacteria and inflammatory factors

**Fig. 4:**
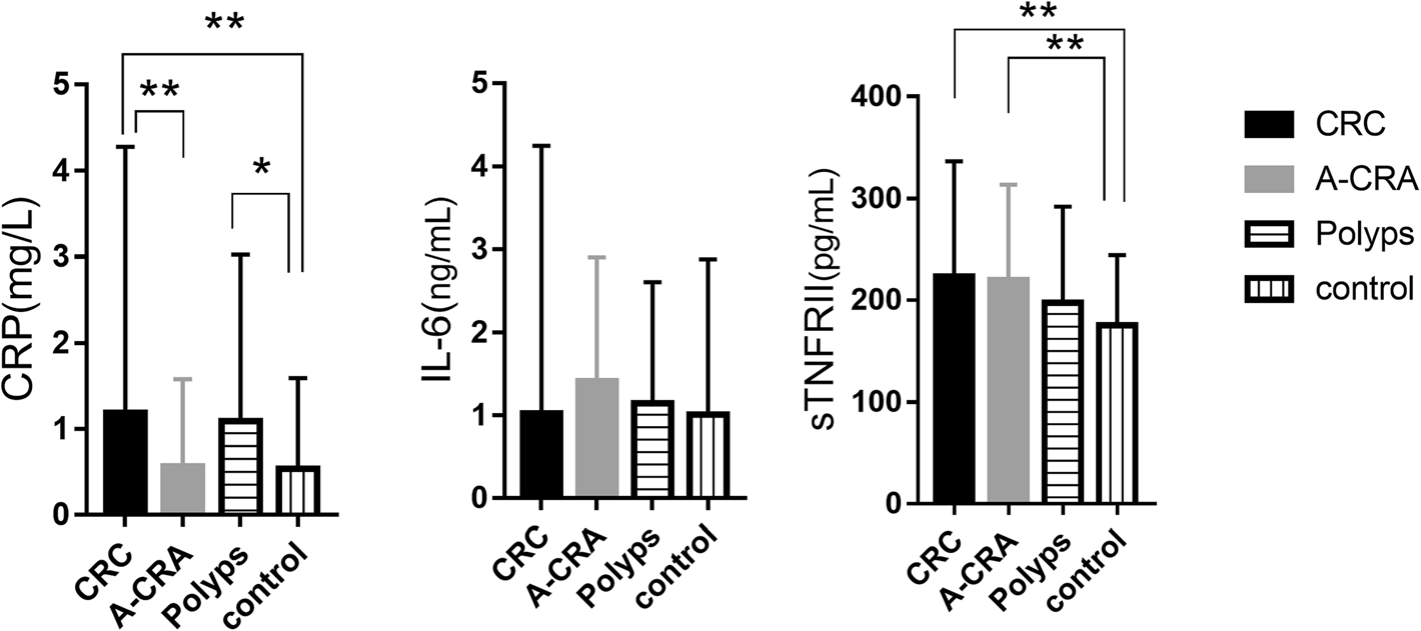
Differences in plasma inflammatory factors among study groups. ** *P* < 0.01, * *P* < 0.05. Kruskal-Wallis tests followed with Dunn’s test post-hoc method. All values are expressed as median ± IQR

We analyzed the evolution of CRC-associated microbiota as the disease progressed. The results showed that all 24 species changed with the order of control-polyps-A-CRA-CRC, but no trend was found with the TNM stage (Additional file 2: Table S1). We also observed that CRP and sTNFR-II increased with the adenoma-carcinoma sequence (Fig. 4, CRP: *JT* = 36,401.5, *P* < 0.001, sTNFR-II: *JT* = 37,225.5, *P* < 0.001).

### Network analysis of CRC-associated microbiota and plasma inflammatory factors

**Fig. 5:**
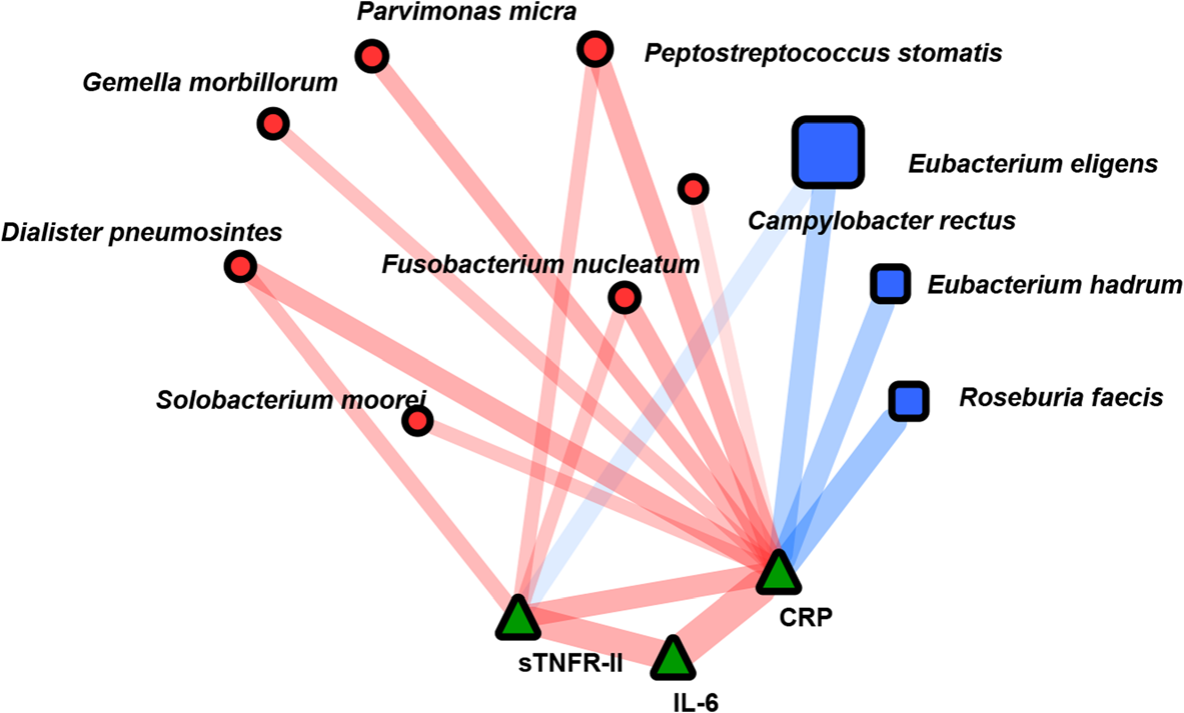
Correlation network among plasma inflammatory factors and CRC-associated species. The width of each edge corresponds to the absolute values of Spearman correlation coefficients and the transparency of edge represents an adjusted *P* value. The line color indicates the direction of a correlation (red for positive and blue for negative). The relative size of the node was determined by the relative abundance of the microbe. Correlations with an adjusted *P* value less than 0.05 were displayed

Data from all the participants were used to analyze the correlations between the CRC-associated microbiota and inflammatory factors (Fig. 5). CRP, IL-6, and sTNFR-II were positively correlated with each other. Seven CRC-associated bacteria that increased in CRC patients including *F. nucleatum*, *P. micra*, *P. stomatis*, *G. morbillorum*, *D. pneumosintes*, *S. moorei* and *C. retus* displayed positive correlations with CRP. *D. pneumosintes*, *F. nucleatum* and *P. stomatis* showed positive correlations with sTNFR-II. Species including *E. eligens*, *E. hadrum* and *R. faecis*, which decreased in CRC patients, showed negative correlations with CRP. *E. eligens* was also negatively correlated with sTNFR-II (Additional file 3: Table S2).

## Discussion

In this case-control study, we identified CRC-associated microbes in CRC patients compared with controls and divided them into two clusters according to the Spearman correlation. Among all the 410 samples from patients and controls, CRC-associated microbes and plasma inflammatory factors changed with the colorectal adenoma-carcinoma sequence. The inflammatory factors were positively correlated with CRC-associated microbes that increased in CRC patients and negatively correlated with those that decreased in CRC patients.

Our results support a previous hypothesis concerning the potent effect of oral periodontopathic bacteria in CRC carcinogenesis [29–31]. As expected, *F. nucleatum* was significantly enriched in CRC patients, which has been discussed widely in previous studies [16, 32, 33]. We also observed increased contributions of periodontal pathogens like *P. micra*, *P. stomatis*, *P. oris*, *D. pneumosintes*. and *C. rectus* [34–37]. The increased abundance of *G. morbillorum* was also observed. *G. morbillorum* is related with endodontic infections [38]. Flynn and his colleagues suggested a polymicrobial synergy hypothesis for the effect of oral pathogens in CRC tumorigenesis [29]. In the periodontitis, after invasive bacteria like *F. nucleatum* disrupt the epithelial barrier, metabolites are produced to change the microenvironment and promote inflammation for latter colonized microbiota like *Porphyromonas* spp. and *Parvimonas* spp. These pathogens can produce harmful factors that interfere with signal pathways, alter the permeability and promote periodontitis by releasing peptides and proteins. A similar mechanism may exist for oral pathogens in the colorectal tumorigenesis. We also observed co-abundance circumstance of these potential pathogens, further suggesting the possibility that they could work mutually in the development of CRC.

Mounting evidence has shown that short-chain fatty acids (SCFAs) acetate, propionate, and butyrate function as suppressors of inflammation and tumorigenesis [5, 39]. The anaerobic microbes inhabited in the large intestine can ferment the undigested dietary components to produce SCFAs. SCFAs can take anti-inflammatory and anti-apoptotic effects by the mechanism including inhibiting histone deacetylase (HDAC) activity [39, 40]. Reducing the expression of inflammatory factors by butyrate via inhibiting the activation of the NF-κB can lead to an anti-inflammatory effect and interfere pre- cancerous cells in the early stage of CRC development [41]. The relationship between changes in SCFAs-producing microbiota and CRC is elucidated in previous studies [31, 42, 43]. Consistent with this notion, our data showed that the relevant abundance of *E. hall II*, *E. hadrum*, *E. desmolans*, *R. faecis* and *C. comes* were depleted, which are butyrate-producing bacteria [44–46]. The commensal bacterium with anti-inflammatory property *S. salivarius* also decreased in CRC patients. As part of the intestinal commensal flora, the reduction in SCFA-producing bacteria may be caused by an increase in other pathogenic microorganisms such as *F. nucleatum*. It is also supported by our observation of negative correlations between potential pathogens and these commensal microbes.

It is widely accepted that most CRC cases are preceded by dysplastic adenomas, and dysplastic adenomas can progress into malignant forms following the adenoma-carcinoma sequence [47, 48]. Previous researches revealed that CRC-associated microbes altered along with the adenoma-carcinoma sequence and suggested the fecal microbiota might be useful in the early diagnosis and treatment of CRC [12, 13]. Consistent with previous studies, in the current study, the potential pathogens and SCFA-producing bacteria tended to increase or decrease with the degree of malignancy, which implied that CRC-associated microbes play an important role in the gradual formation of the tumor microenvironment.

TNF-α and IL-6 are core cytokines in the colorectal tumor promotion by activating NF-κB signaling pathways and STAT3 [5, 49, 50]. CRP can be produced by hepatocytes in response to the IL-6 activated by immune cells [11] and can also enhance resistance to apoptosis via STAT3 and NF-κB pathway [51–53]. Experimental studies have revealed that inflammatory factors play an important role in the relationship between pathogens like *F. nucleatum* and CRC [20, 54]. Elevation of related gene expression and activation of pathways are also observed in CRC patients [11, 55]. However, previous population-based studies did not support inflammatory factors (IL-6, CRP, and TNF-α) to be adequate biomarkers for CRC [14, 17]. In our study, CRP and sTNFR-II tended to increase along the adenoma-carcinoma sequence. It is intriguing that, among all the participants, CRP and sTNFR-II were significantly correlated with several CRC-associated microbes. However, no significant difference was observed in IL-6 among the four study groups and no association was observed between plasma IL-6 and bacteria. The level of IL-6 in plasma is also influenced by adiposity and inflammation of other tissues which might be the reason for the inconsistency with CRP and sTNFR-II [14]. Further human and experimental research is warranted to confirm this relationship and disentangle the complex role of immune response in CRC.

To our knowledge, this is the first case-control study to explore the associations of plasma inflammatory factors with CRC-associated microbiota. The enrolled patients were unaware of their disease status at the time of sampling and stool samples were obtained before colonoscopy, which avoided potential impacts of lifestyle and dietary changes. Strict operations conducted within one hospital reduced potential diagnostic biases.

There were some limitations in our study. Only stool samples rather than mucosal specimen were used to study the profiles of the gut microbiome. Stool samples are considered to be only partially uniform with those of the mucosal microbiota [11, 56]. Repeated measurements and external validation could improve the stability and credibility of data but were not conducted in the current study. In addition, we chose 16S rDNA amplicon sequencing considering the cost and sample size, which is less accurate compared with the shotgun sequencing method, especially at the species level.

## Conclusions

The current study investigated CRC-associated microbiota and their correlations with inflammatory factors. These bacteria, as well as plasma CRP and sTNFR-II, changed along the adenoma-carcinoma sequence. Several microbes were significantly correlated with CRP and sTNFR-II. Our study results support the note that gut microbiome and inflammation may gradually form a microenvironment to promote the development of CRC.

## Additional files

Additional file 1: **Figure S1.** PCOA analysis based on unweighted and weighted Unifrac distances. Figure S1A and B are PCOA results based on the unweighted Unifrac distance. Figure S1C and D are PCOA results based on the weighted Unifrac distance. (TIF 4739 kb)

Additional file 2: **Table S1.** The mean relative abundance, statistical parameter and trend analysis of CRC-associated microbes. (DOCX 20 kb)

Additional file 3: **Table S2.** Correlations among CRC-associated microbes and plasma inflammatory factors. (DOCX 31 kb)

## Abbreviations

A-CRA: Advanced colorectal adenoma
BMI: Body mass index
CRC: Colorectal cancer
CRP: C-reactive protein
ETBF: Enterotoxigenic *B. fragilis*
IL-6: Interleukin 6
OTU: Operational taxonomic units
PCoA: Principal Coordinates Analysis
PCR: Polymerase chain reaction
PMANOVA: Permutational Multivariate Analysis of Variance Using Distance Matrices
STAT3: Signal transducer and activator of transcription 3
sTNFR-II: Soluble tumor necrosis factor II
TNF-α: Tumor necrosis factor
