# Supplementary figures and images for: Changes in gut microbiota and plasma inflammatory factors across the stages of colorectal tumorigenesis: a case-control study
Source: BMC Microbiol. 2018 Aug 29;18:92. doi: 10.1186/s12866-018-1232-6 (PMC6114884; doi:10.1186/s12866-018-1232-6)

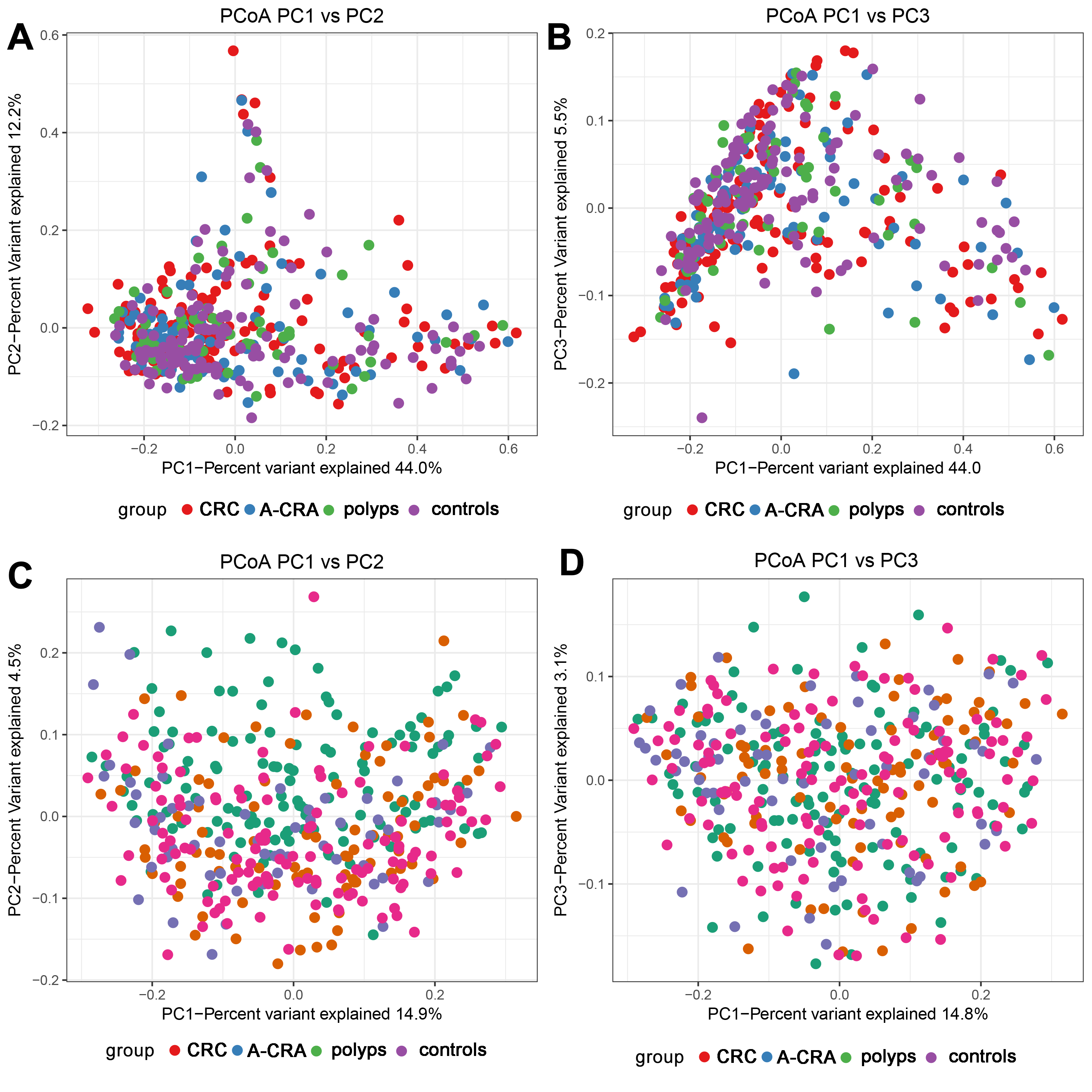

Supplement: Supplementary file 1 — Figure S1. PCOA analysis based on unweighted and weighted Unifrac distances. Figure S1A and B are PCOA results based on the unweighted Unifrac distance. Figure S1C and D are PCOA results based on the weighted Unifrac distance. (TIF 4739 kb) [file 12866_2018_1232_MOESM1_ESM.tif]
