# Supplementary material for: Changes in gut microbiota and plasma inflammatory factors across the stages of colorectal tumorigenesis: a case-control study
Source: BMC Microbiol. 2018 Aug 29;18:92. doi: 10.1186/s12866-018-1232-6 (PMC6114884; doi:10.1186/s12866-018-1232-6)
Supplement: Supplementary file 2 — Table S1. The mean relative abundance, statistical parameter and trend analysis of CRC-associated microbes. (DOCX 20 kb) [file 12866_2018_1232_MOESM2_ESM.docx]

Table S1. The mean relative abundance, statistical parameter and trend analysis of CRC-associated microbes.

| **Species** | **Mean relevance abundance (%)** | | | | **Mean Importance*** | **normHits*** | **Log FC** | **adjusted *P* value※** | **cluster** | ***P_trend_* §** | ***P_trend_* †** |
| --- | --- | --- | --- | --- | --- | --- | --- | --- | --- | --- | --- |
|  | **CRC** | **A-CRA** | **polyps** | **control** |  |  |  |  |  |  |  |
| *Eubacterium eligens* | 0.484 | 1.042 | 1.277 | 1.791 | 8.802 | 1.00 | -1.53 | < 0.001 | 1 | < 0.001 | 0.886 |
| *Coprococcus comes* | 0.095 | 0.130 | 0.109 | 0.158 | 2.971 | 0.57 | -0.94 | < 0.001 | 1 | 0.001 | 0.749 |
| *Eubacterium hadrum* | 0.167 | 0.160 | 0.336 | 0.352 | 6.631 | 0.99 | -0.93 | 0.001 | 1 | < 0.001 | 0.292 |
| *Eubacterium hallii* | 0.178 | 0.218 | 0.190 | 0.219 | 7.709 | 0.99 | -0.72 | 0.002 | 1 | 0.001 | 0.678 |
| *Fusicatenibacter saccharivorans* | 0.238 | 0.247 | 0.200 | 0.316 | 2.940 | 0.56 | -0.79 | 0.003 | 1 | < 0.001 | 0.620 |
| *Blautia faecis* | 0.175 | 0.208 | 0.190 | 0.223 | 3.545 | 0.72 | -0.57 | 0.004 | 1 | 0.015 | 0.277 |
| *Roseburia faecis* | 0.233 | 0.241 | 0.205 | 0.314 | 2.569 | 0.47 | -0.95 | 0.005 | 1 | < 0.001 | 0.152 |
| *Ruminococcus lactaris* | 0.051 | 0.049 | 0.060 | 0.108 | 2.918 | 0.56 | -1.04 | 0.006 | 1 | 0.001 | 0.733 |
| *Eubacterium desmolans* | 0.249 | 0.323 | 0.316 | 0.341 | 3.465 | 0.69 | -0.48 | 0.019 | 1 | 0.003 | 0.517 |
| *Clostridium lactatifermentans* | 0.705 | 0.370 | 0.211 | 0.280 | 3.235 | 0.64 | 0.64 | 0.022 | 1 | 0.001 | 0.690 |
| *Streptococcus salivarius* | 0.253 | 0.454 | 0.593 | 0.392 | 3.264 | 0.65 | -0.71 | 0.035 | 1 | < 0.001 | 0.290 |
| *Peptostreptococcus stomatis* | 0.455 | 0.009 | 0.023 | 0.005 | 18.652 | 1.00 | 3.37 | < 0.001 | 2 | < 0.001 | 0.812 |
| *Parvimonas micra* | 0.240 | 0.008 | 0.012 | 0.002 | 21.186 | 1.00 | 3.67 | < 0.001 | 2 | < 0.001 | 0.500 |
| *Gemella morbillorum* | 0.073 | 0.008 | 0.007 | 0.026 | 7.547 | 1.00 | 1.41 | < 0.001 | 2 | < 0.001 | 0.459 |
| *Dialister pneumosintes* | 0.141 | 0.001 | 0.002 | 0.001 | 13.307 | 1.00 | 3.76 | < 0.001 | 2 | < 0.001 | 0.390 |
| *Porphyromonas asaccharolytica* | 0.709 | 0.010 | 0.004 | 0.007 | 8.730 | 1.00 | 3.67 | < 0.001 | 2 | < 0.001 | 0.645 |
| *Solobacterium moorei* | 0.028 | 0.002 | 0.003 | 0.006 | 4.921 | 0.94 | 1.49 | < 0.001 | 2 | < 0.001 | 0.262 |
| *Eisenbergiella tayi* | 0.159 | 0.115 | 0.029 | 0.041 | 2.923 | 0.58 | 1.27 | < 0.001 | 2 | < 0.001 | 0.645 |
| *Fusobacterium nucleatum* | 0.170 | 0.003 | 0.002 | 0.001 | 6.766 | 0.99 | 2.35 | < 0.001 | 2 | < 0.001 | 0.789 |
| *Ruminococcus torques* | 0.574 | 0.398 | 0.286 | 0.282 | 5.136 | 0.94 | 0.95 | < 0.001 | 2 | < 0.001 | 0.797 |
| *Eggerthella lenta* | 0.019 | 0.009 | 0.009 | 0.008 | 3.165 | 0.64 | 0.79 | 0.003 | 2 | < 0.001 | 0.927 |
| *Clostridium symbiosum* | 0.024 | 0.013 | 0.009 | 0.007 | 2.736 | 0.50 | 1.09 | 0.004 | 2 | < 0.001 | 0.468 |
| *Campylobacter rectus* | 0.005 | < 0.001 | < 0.001 | < 0.001 | 3.040 | 0.61 | 2.19 | 0.014 | 2 | < 0.001 | 0.132 |
| *Clostridium scindens* | 0.021 | 0.019 | 0.015 | 0.006 | 3.057 | 0.59 | 0.85 | 0.037 | 2 | < 0.001 | 0.352 |

※: adjusted *P* values are calculated by Zero-inflated Log-Normal mixture model and adjusted using Benjamini and Hochberg method.

*: Mean importance and normHits are the coefficients from random forest algorithm and the larger the number, the more valuable the bacteria.

§: *P* values for trend analysis of the order of controls–polyps–A-CRA–CRC. †: *P* values for trend analysis of TNM stage;

Log FC was calculated by Zero-inflated Log-Normal mixture model in metagenomeSeq package.
