# Supplementary material for: Changes in gut microbiota and plasma inflammatory factors across the stages of colorectal tumorigenesis: a case-control study
Source: BMC Microbiol. 2018 Aug 29;18:92. doi: 10.1186/s12866-018-1232-6 (PMC6114884; doi:10.1186/s12866-018-1232-6)
Supplement: Supplementary file 3 — Table S2. Correlations among CRC-associated microbes and plasma inflammatory factors. (DOCX 31 kb) [file 12866_2018_1232_MOESM3_ESM.docx]

**Table S2**. Correlations among CRC-associated microbes and plasma inflammatory factrs.

| **Factor1** | **Factor2** | **r** | **lower CI**  **(95%)** | **Higher CI**  **(95%)** | ***P* value** |
| --- | --- | --- | --- | --- | --- |
| CRP | IL-6 | 0.369 | 0.283 | 0.450 | < 0.001 |
| CRP | STNFR-Ⅱ | 0.229 | 0.135 | 0.319 | < 0.001 |
| IL-6 | STNFR-Ⅱ | 0.304 | 0.214 | 0.390 | < 0.001 |
| CRP | *Dialister pneumosintes* | 0.231 | 0.137 | 0.321 | < 0.001 |
| CRP | *Peptostreptococcus stomatis* | 0.214 | 0.119 | 0.304 | < 0.001 |
| CRP | *Fusobacterium nucleatum* | 0.201 | 0.106 | 0.292 | < 0.001 |
| CRP | *Parvimonas micra* | 0.168 | 0.072 | 0.261 | 0.002 |
| CRP | *Roseburia faecis* | -0.160 | -0.253 | -0.064 | 0.003 |
| CRP | *Eubacterium hadrum* | -0.136 | -0.230 | -0.040 | 0.014 |
| CRP | *Eubacterium eligens* | -0.134 | -0.228 | -0.038 | 0.015 |
| CRP | *Gemella morbillorum* | 0.134 | 0.037 | 0.228 | 0.016 |
| CRP | *Solobacterium moorei* | 0.131 | 0.035 | 0.225 | 0.018 |
| CRP | *Campylobacter rectus* | 0.114 | 0.017 | 0.209 | 0.042 |
| STNFR-Ⅱ | *Dialister pneumosintes* | 0.141 | 0.045 | 0.235 | 0.010 |
| STNFR-Ⅱ | *Peptostreptococcus stomatis* | 0.133 | 0.037 | 0.227 | 0.016 |
| STNFR-Ⅱ | *Fusobacterium nucleatum* | 0.130 | 0.034 | 0.224 | 0.018 |
| STNFR-Ⅱ | *Eubacterium eligens* | -0.111 | -0.205 | -0.014 | 0.049 |
| *Peptostreptococcus stomatis* | *Parvimonas micra* | 0.710 | 0.658 | 0.755 | < 0.001 |
| *Peptostreptococcus stomatis* | *Dialister pneumosintes* | 0.610 | 0.546 | 0.668 | < 0.001 |
| *Peptostreptococcus stomatis* | *Gemella morbillorum* | 0.474 | 0.396 | 0.546 | < 0.001 |
| *Peptostreptococcus stomatis* | *Porphyromonas asaccharolytica* | 0.430 | 0.347 | 0.505 | < 0.001 |
| *Peptostreptococcus stomatis* | *Solobacterium moorei* | 0.407 | 0.323 | 0.485 | < 0.001 |
| *Peptostreptococcus stomatis* | *Fusobacterium nucleatum* | 0.398 | 0.314 | 0.477 | < 0.001 |
| *Peptostreptococcus stomatis* | *Campylobacter rectus* | 0.358 | 0.270 | 0.439 | < 0.001 |
| *Peptostreptococcus stomatis* | *Clostridium lactatifermentans* | 0.236 | 0.142 | 0.325 | < 0.001 |
| *Peptostreptococcus stomatis* | *Ruminococcus torques* | 0.226 | 0.132 | 0.316 | < 0.001 |
| *Peptostreptococcus stomatis* | *Eubacterium eligens* | -0.211 | -0.302 | -0.117 | < 0.001 |
| *Peptostreptococcus stomatis* | *Clostridium scindens* | 0.198 | 0.103 | 0.290 | < 0.001 |
| *Peptostreptococcus stomatis* | *Eisenbergiella tayi* | 0.198 | 0.103 | 0.289 | < 0.001 |
| *Peptostreptococcus stomatis* | *Eubacterium desmolans* | -0.174 | -0.266 | -0.078 | 0.001 |
| *Peptostreptococcus stomatis* | *Eubacterium hadrum* | -0.168 | -0.261 | -0.073 | 0.002 |
| *Peptostreptococcus stomatis* | *Clostridium symbiosum* | 0.161 | 0.066 | 0.254 | 0.003 |
| *Peptostreptococcus stomatis* | *Roseburia faecis* | -0.161 | -0.254 | -0.065 | 0.003 |
| *Peptostreptococcus stomatis* | *Fusicatenibacter saccharivorans* | -0.129 | -0.223 | -0.032 | 0.020 |
| *Parvimonas micra* | *Dialister pneumosintes* | 0.611 | 0.546 | 0.668 | < 0.001 |
| *Parvimonas micra* | *Porphyromonas asaccharolytica* | 0.491 | 0.414 | 0.561 | < 0.001 |
| *Parvimonas micra* | *Gemella morbillorum* | 0.453 | 0.373 | 0.527 | < 0.001 |
| *Parvimonas micra* | *Fusobacterium nucleatum* | 0.423 | 0.340 | 0.499 | < 0.001 |

| *Parvimonas micra* | *Solobacterium moorei* | 0.386 | 0.300 | 0.465 | < 0.001 |
| --- | --- | --- | --- | --- | --- |
| *Parvimonas micra* | *Campylobacter rectus* | 0.317 | 0.227 | 0.401 | < 0.001 |
| *Parvimonas micra* | *Eisenbergiella tayi* | 0.304 | 0.214 | 0.390 | < 0.001 |
| *Parvimonas micra* | *Clostridium lactatifermentans* | 0.288 | 0.197 | 0.375 | < 0.001 |
| *Parvimonas micra* | *Ruminococcus torques* | 0.273 | 0.181 | 0.361 | < 0.001 |
| *Parvimonas micra* | *Clostridium scindens* | 0.261 | 0.168 | 0.349 | < 0.001 |
| *Parvimonas micra* | *Clostridium symbiosum* | 0.209 | 0.115 | 0.300 | < 0.001 |
| *Parvimonas micra* | *Eubacterium eligens* | -0.177 | -0.270 | -0.082 | 0.001 |
| *Parvimonas micra* | *Streptococcus salivarius* | -0.139 | -0.233 | -0.043 | 0.012 |
| *Parvimonas micra* | *Eggerthella lenta* | 0.120 | 0.023 | 0.214 | 0.032 |
| *Parvimonas micra* | *Eubacterium hadrum* | -0.119 | -0.213 | -0.022 | 0.034 |
| *Eubacterium eligens* | *Roseburia faecis* | 0.482 | 0.404 | 0.553 | < 0.001 |
| *Eubacterium eligens* | *Blautia faecis* | 0.351 | 0.263 | 0.433 | < 0.001 |
| *Eubacterium eligens* | *Fusicatenibacter saccharivorans* | 0.344 | 0.255 | 0.426 | < 0.001 |
| *Eubacterium eligens* | *Ruminococcus lactaris* | 0.304 | 0.213 | 0.389 | < 0.001 |
| *Eubacterium eligens* | *Eubacterium desmolans* | 0.280 | 0.188 | 0.367 | < 0.001 |
| *Eubacterium eligens* | *Eubacterium hallii* | 0.263 | 0.171 | 0.351 | < 0.001 |
| *Eubacterium eligens* | *Eubacterium hadrum* | 0.250 | 0.157 | 0.338 | < 0.001 |
| *Eubacterium eligens* | *Coprococcus comes* | 0.250 | 0.157 | 0.338 | < 0.001 |
| *Eubacterium eligens* | *Eggerthella lenta* | -0.189 | -0.281 | -0.094 | < 0.001 |
| *Eubacterium eligens* | *Gemella morbillorum* | -0.166 | -0.259 | -0.071 | 0.002 |
| *Eubacterium eligens* | *Clostridium symbiosum* | -0.151 | -0.244 | -0.055 | 0.006 |
| *Eubacterium eligens* | *Solobacterium moorei* | -0.135 | -0.229 | -0.039 | 0.015 |
| *Eubacterium eligens* | *Eisenbergiella tayi* | -0.135 | -0.229 | -0.038 | 0.015 |
| *Eubacterium eligens* | *Clostridium lactatifermentans* | 0.124 | 0.028 | 0.219 | 0.026 |
| *Eubacterium eligens* | *Fusobacterium nucleatum* | -0.115 | -0.210 | -0.019 | 0.039 |
| *Gemella morbillorum* | *Dialister pneumosintes* | 0.431 | 0.348 | 0.507 | < 0.001 |
| *Gemella morbillorum* | *Solobacterium moorei* | 0.389 | 0.303 | 0.468 | < 0.001 |
| *Gemella morbillorum* | *Fusobacterium nucleatum* | 0.349 | 0.261 | 0.431 | < 0.001 |
| *Gemella morbillorum* | *Campylobacter rectus* | 0.302 | 0.211 | 0.387 | < 0.001 |
| *Gemella morbillorum* | *Porphyromonas asaccharolytica* | 0.241 | 0.148 | 0.330 | < 0.001 |
| *Gemella morbillorum* | *Streptococcus salivarius* | 0.165 | 0.069 | 0.258 | 0.002 |
| *Gemella morbillorum* | *Clostridium lactatifermentans* | 0.133 | 0.037 | 0.227 | 0.016 |
| *Gemella morbillorum* | *Fusicatenibacter saccharivorans* | -0.124 | -0.218 | -0.027 | 0.026 |
| *Gemella morbillorum* | *Roseburia faecis* | -0.117 | -0.211 | -0.020 | 0.037 |
| *Dialister pneumosintes* | *Fusobacterium nucleatum* | 0.452 | 0.372 | 0.526 | < 0.001 |
| *Dialister pneumosintes* | *Solobacterium moorei* | 0.372 | 0.285 | 0.453 | < 0.001 |
| *Dialister pneumosintes* | *Porphyromonas asaccharolytica* | 0.358 | 0.271 | 0.440 | < 0.001 |
| *Dialister pneumosintes* | *Campylobacter rectus* | 0.273 | 0.181 | 0.360 | < 0.001 |
| *Dialister pneumosintes* | *Clostridium lactatifermentans* | 0.217 | 0.122 | 0.307 | < 0.001 |
| *Dialister pneumosintes* | *Clostridium scindens* | 0.206 | 0.112 | 0.297 | < 0.001 |
| *Dialister pneumosintes* | *Eisenbergiella tayi* | 0.202 | 0.107 | 0.293 | < 0.001 |
| *Dialister pneumosintes* | *Ruminococcus torques* | 0.185 | 0.090 | 0.277 | < 0.001 |
| *Dialister pneumosintes* | *Clostridium symbiosum* | 0.149 | 0.053 | 0.242 | 0.006 |

| *Dialister pneumosintes* | *Eubacterium desmolans* | -0.136 | -0.230 | -0.040 | 0.014 |
| --- | --- | --- | --- | --- | --- |
| *Porphyromonas asaccharolytica* | *Eisenbergiella tayi* | 0.305 | 0.214 | 0.390 | < 0.001 |
| *Porphyromonas asaccharolytica* | *Clostridium lactatifermentans* | 0.281 | 0.189 | 0.368 | < 0.001 |
| *Porphyromonas asaccharolytica* | *Solobacterium moorei* | 0.245 | 0.152 | 0.334 | < 0.001 |
| *Porphyromonas asaccharolytica* | *Fusobacterium nucleatum* | 0.216 | 0.122 | 0.307 | < 0.001 |
| *Porphyromonas asaccharolytica* | *Ruminococcus torques* | 0.199 | 0.104 | 0.290 | < 0.001 |
| *Porphyromonas asaccharolytica* | *Campylobacter rectus* | 0.134 | 0.037 | 0.228 | 0.016 |
| *Porphyromonas asaccharolytica* | *Clostridium scindens* | 0.118 | 0.021 | 0.212 | 0.036 |
| *Porphyromonas asaccharolytica* | *Coprococcus comes* | 0.115 | 0.019 | 0.210 | 0.039 |
| *Solobacterium moorei* | *Fusobacterium nucleatum* | 0.290 | 0.199 | 0.376 | < 0.001 |
| *Solobacterium moorei* | *Clostridium scindens* | 0.174 | 0.078 | 0.266 | 0.001 |
| *Solobacterium moorei* | *Roseburia faecis* | -0.136 | -0.230 | -0.040 | 0.014 |
| *Solobacterium moorei* | *Eubacterium desmolans* | -0.122 | -0.216 | -0.025 | 0.029 |
| *Solobacterium moorei* | *Streptococcus salivarius* | 0.121 | 0.024 | 0.215 | 0.031 |
| *Coprococcus comes* | *Ruminococcus lactaris* | 0.344 | 0.255 | 0.426 | < 0.001 |
| *Coprococcus comes* | *Eubacterium hallii* | 0.343 | 0.254 | 0.426 | < 0.001 |
| *Coprococcus comes* | *Blautia faecis* | 0.308 | 0.218 | 0.393 | < 0.001 |
| *Coprococcus comes* | *Fusicatenibacter saccharivorans* | 0.288 | 0.197 | 0.374 | < 0.001 |
| *Coprococcus comes* | *Eubacterium desmolans* | 0.258 | 0.166 | 0.347 | < 0.001 |
| *Coprococcus comes* | *Roseburia faecis* | 0.240 | 0.147 | 0.329 | < 0.001 |
| *Coprococcus comes* | *Clostridium symbiosum* | -0.238 | -0.327 | -0.145 | < 0.001 |
| *Coprococcus comes* | *Clostridium lactatifermentans* | 0.232 | 0.138 | 0.321 | < 0.001 |
| *Coprococcus comes* | *Eubacterium hadrum* | 0.217 | 0.123 | 0.308 | < 0.001 |
| *Coprococcus comes* | *Streptococcus salivarius* | 0.191 | 0.096 | 0.283 | < 0.001 |
| *Coprococcus comes* | *Ruminococcus torques* | 0.132 | 0.036 | 0.226 | 0.017 |
| *Coprococcus comes* | *Eggerthella lenta* | -0.122 | -0.216 | -0.025 | 0.029 |
| *Eisenbergiella tayi* | *Ruminococcus torques* | 0.415 | 0.331 | 0.492 | < 0.001 |
| *Eisenbergiella tayi* | *Clostridium scindens* | 0.361 | 0.273 | 0.442 | < 0.001 |
| *Eisenbergiella tayi* | *Clostridium symbiosum* | 0.357 | 0.269 | 0.439 | < 0.001 |
| *Eisenbergiella tayi* | *Clostridium lactatifermentans* | 0.291 | 0.200 | 0.378 | < 0.001 |
| *Eisenbergiella tayi* | *Eggerthella lenta* | 0.227 | 0.133 | 0.317 | < 0.001 |
| *Eisenbergiella tayi* | *Blautia faecis* | 0.194 | 0.099 | 0.285 | < 0.001 |
| *Eisenbergiella tayi* | *Streptococcus salivarius* | -0.160 | -0.252 | -0.064 | 0.003 |
| *Eisenbergiella tayi* | *Roseburia faecis* | -0.141 | -0.235 | -0.045 | 0.010 |
| *Ruminococcus torques* | *Clostridium scindens* | 0.362 | 0.274 | 0.443 | < 0.001 |
| *Ruminococcus torques* | *Clostridium lactatifermentans* | 0.310 | 0.220 | 0.395 | < 0.001 |
| *Ruminococcus torques* | *Fusobacterium nucleatum* | 0.214 | 0.119 | 0.304 | < 0.001 |
| *Ruminococcus torques* | *Eggerthella lenta* | 0.213 | 0.118 | 0.303 | < 0.001 |
| *Ruminococcus torques* | *Eubacterium hallii* | 0.213 | 0.118 | 0.303 | < 0.001 |
| *Ruminococcus torques* | *Clostridium symbiosum* | 0.196 | 0.101 | 0.287 | < 0.001 |
| *Ruminococcus torques* | *Blautia faecis* | 0.187 | 0.092 | 0.279 | < 0.001 |
| *Ruminococcus torques* | *Ruminococcus lactaris* | 0.149 | 0.052 | 0.242 | 0.007 |
| *Ruminococcus torques* | *Eubacterium desmolans* | 0.139 | 0.043 | 0.233 | 0.012 |

| *Ruminococcus torques* | *Streptococcus salivarius* | -0.111 | -0.205 | -0.014 | 0.049 |
| --- | --- | --- | --- | --- | --- |
| *Fusobacterium nucleatum* | *Campylobacter rectus* | 0.264 | 0.171 | 0.351 | < 0.001 |
| *Fusobacterium nucleatum* | *Eggerthella lenta* | 0.164 | 0.068 | 0.257 | 0.002 |
| *Fusobacterium nucleatum* | *Clostridium lactatifermentans* | 0.157 | 0.061 | 0.250 | 0.004 |
| *Fusobacterium nucleatum* | *Clostridium scindens* | 0.147 | 0.051 | 0.240 | 0.007 |
| *Fusobacterium nucleatum* | *Clostridium symbiosum* | 0.116 | 0.019 | 0.210 | 0.038 |
| *Fusobacterium nucleatum* | *Eubacterium hadrum* | -0.114 | -0.208 | -0.017 | 0.042 |
| *Eubacterium hadrum* | *Eubacterium hallii* | 0.532 | 0.459 | 0.598 | < 0.001 |
| *Eubacterium hadrum* | *Fusicatenibacter saccharivorans* | 0.435 | 0.353 | 0.511 | < 0.001 |
| *Eubacterium hadrum* | *Eubacterium desmolans* | 0.375 | 0.289 | 0.456 | < 0.001 |
| *Eubacterium hadrum* | *Blautia faecis* | 0.323 | 0.234 | 0.407 | < 0.001 |
| *Eubacterium hadrum* | *Ruminococcus lactaris* | 0.279 | 0.187 | 0.366 | < 0.001 |
| *Eubacterium hadrum* | *Roseburia faecis* | 0.212 | 0.118 | 0.303 | < 0.001 |
| *Eubacterium hadrum* | *Streptococcus salivarius* | 0.196 | 0.101 | 0.287 | < 0.001 |
| *Eubacterium hadrum* | *Campylobacter rectus* | -0.172 | -0.264 | -0.076 | 0.001 |
| *Eubacterium hadrum* | *Clostridium lactatifermentans* | 0.160 | 0.064 | 0.253 | 0.003 |
| *Eubacterium hadrum* | *Clostridium symbiosum* | -0.157 | -0.250 | -0.061 | 0.004 |
| *Eubacterium hallii* | *Fusicatenibacter saccharivorans* | 0.474 | 0.395 | 0.545 | < 0.001 |
| *Eubacterium hallii* | *Blautia faecis* | 0.452 | 0.371 | 0.525 | < 0.001 |
| *Eubacterium hallii* | *Eubacterium desmolans* | 0.350 | 0.262 | 0.432 | < 0.001 |
| *Eubacterium hallii* | *Clostridium lactatifermentans* | 0.323 | 0.233 | 0.407 | < 0.001 |
| *Eubacterium hallii* | *Ruminococcus lactaris* | 0.319 | 0.229 | 0.403 | < 0.001 |
| *Eubacterium hallii* | *Roseburia faecis* | 0.310 | 0.220 | 0.395 | < 0.001 |
| *Eubacterium hallii* | *Streptococcus salivarius* | 0.150 | 0.054 | 0.244 | 0.006 |
| *Eubacterium hallii* | *Clostridium symbiosum* | -0.144 | -0.238 | -0.048 | 0.008 |
| *Fusicatenibacter saccharivorans* | *Roseburia faecis* | 0.437 | 0.355 | 0.512 | < 0.001 |
| *Fusicatenibacter saccharivorans* | *Blautia faecis* | 0.388 | 0.303 | 0.467 | < 0.001 |
| *Fusicatenibacter saccharivorans* | *Ruminococcus lactaris* | 0.300 | 0.209 | 0.386 | < 0.001 |
| *Fusicatenibacter saccharivorans* | *Eubacterium desmolans* | 0.295 | 0.204 | 0.381 | < 0.001 |
| *Fusicatenibacter saccharivorans* | *Clostridium lactatifermentans* | 0.226 | 0.132 | 0.316 | < 0.001 |
| *Fusicatenibacter saccharivorans* | *Streptococcus salivarius* | 0.218 | 0.124 | 0.309 | < 0.001 |
| *Fusicatenibacter saccharivorans* | *Clostridium symbiosum* | -0.186 | -0.278 | -0.091 | < 0.001 |
| *Eggerthella lenta* | *Clostridium scindens* | 0.344 | 0.256 | 0.427 | < 0.001 |
| *Eggerthella lenta* | *Clostridium symbiosum* | 0.310 | 0.219 | 0.395 | < 0.001 |
| *Eggerthella lenta* | *Roseburia faecis* | -0.181 | -0.273 | -0.086 | 0.001 |
| *Clostridium symbiosum* | *Clostridium scindens* | 0.295 | 0.204 | 0.381 | < 0.001 |
| *Clostridium symbiosum* | *Roseburia faecis* | -0.192 | -0.283 | -0.097 | < 0.001 |
| *Clostridium symbiosum* | *Streptococcus salivarius* | -0.132 | -0.226 | -0.035 | 0.017 |
| *Clostridium symbiosum* | *Campylobacter rectus* | 0.117 | 0.020 | 0.211 | 0.037 |
| *Blautia faecis* | *Clostridium lactatifermentans* | 0.355 | 0.267 | 0.437 | < 0.001 |
| *Blautia faecis* | *Roseburia faecis* | 0.349 | 0.261 | 0.432 | < 0.001 |
| *Blautia faecis* | *Eubacterium desmolans* | 0.300 | 0.209 | 0.385 | < 0.001 |
| *Blautia faecis* | *Ruminococcus lactaris* | 0.278 | 0.186 | 0.365 | < 0.001 |

| *Blautia faecis* | *Clostridium scindens* | 0.130 | 0.033 | 0.224 | 0.019 |
| --- | --- | --- | --- | --- | --- |
| *Roseburia faecis* | *Ruminococcus lactaris* | 0.232 | 0.138 | 0.321 | < 0.001 |
| *Roseburia faecis* | *Eubacterium desmolans* | 0.204 | 0.109 | 0.295 | < 0.001 |
| *Roseburia faecis* | *Clostridium lactatifermentans* | 0.167 | 0.071 | 0.260 | 0.002 |
| *Roseburia faecis* | *Streptococcus salivarius* | 0.118 | 0.021 | 0.212 | 0.035 |
| *Ruminococcus lactaris* | *Clostridium lactatifermentans* | 0.220 | 0.126 | 0.310 | < 0.001 |
| *Ruminococcus lactaris* | *Eubacterium desmolans* | 0.190 | 0.094 | 0.281 | < 0.001 |
| *Campylobacter rectus* | *Clostridium lactatifermentans* | 0.185 | 0.090 | 0.277 | < 0.001 |
| *Eubacterium desmolans* | *Clostridium lactatifermentans* | 0.207 | 0.113 | 0.298 | < 0.001 |
| *Eubacterium desmolans* | *Streptococcus salivarius* | 0.149 | 0.053 | 0.242 | 0.006 |
| *Clostridium lactatifermentans* | *Clostridium scindens* | 0.122 | 0.025 | 0.216 | 0.029 |

* r, 95% CIs and adjusted *P* Values were calculated by spearman correlation tests. *P* Values were adjusted by FDR method.
